# Redescription, Morphogenesis, and Molecular Phylogeny of *Pseudosincirra longicirrata* nov. comb., With Establishment of a New Genus *Pseudosincirra* nov. gen. (Ciliophora, Hypotrichia)

**DOI:** 10.3389/fmicb.2021.777540

**Published:** 2021-11-30

**Authors:** Qi Gao, Chen Shao, Qiuyue Tang, Jingbao Li

**Affiliations:** ^1^Laboratory of Protozoological Biodiversity and Evolution in Wetland, College of Life Sciences, Shaanxi Normal University, Xi’an, China; ^2^Key Laboratory for Space Bioscience and Biotechnology, Institute of Special Environmental Biophysics, School of Life Sciences, Northwestern Polytechnical University, Xi’an, China

**Keywords:** Hypotrichia, morphogenesis, new genus, phylogenetic analyses, *Pseudosincirra* nov. gen.

## Abstract

The morphology and morphogenesis of *Pseudosincirra longicirrata* nov. gen. and nov. comb., isolated from southern China, were investigated with living observation and protargol staining. Our population is similar to the original population in living characteristics and ciliary patterns. The main determinable morphogenetic features of *P. longicirrata* nov. comb. are the presence of five frontoventral-transverse cirral anlagen (FVT-anlagen) and a dorsomarginal kinety anlage. According to the origin of FVT-anlagen IV and V in proter, it can be determined that *P. longicirrata* nov. comb. possesses two frontoventral rows and one right marginal row. Hence, a new genus, *Pseudosincirra* nov. gen., is proposed, and the diagnosis of *P. longicirrata* nov. comb. is improved. The new genus is diagnosed as follows: adoral zone of membranelles and undulating membranes is in a *Gonostomum* pattern; there are three enlarged frontal cirri, one buccal cirrus, and one parabuccal cirrus; postperistomial cirrus and transverse cirri are lacking; there are two more or less long frontoventral rows and one right and two or more left marginal rows; cirri within all rows very widely spaced; dorsal kinety pattern is of *Urosomoida* type, that is, three dorsal kineties and one dorsomarginal kinety; and caudal cirri are present. Phylogenetic analyses based on the small subunit ribosomal (SSU rDNA) sequence data indicate that *P. longicirrata* nov. comb. clusters with *Deviata* and *Perisincirra*. It is considered that *Pseudosincirra* nov. gen. and *Perisincirra paucicirrata* should be assigned to the family Deviatidae; fine cirri, and cirri within all rows being relatively widely spaced, should be considered as plesiomorphies of Deviatidae; and Deviatidae is closely related to Dorsomarginalia or *Strongylidium*–*Hemiamphisiella*–*Pseudouroleptus.*

## Introduction

Ciliates are a large group of unicellular eukaryotes, many of which have cosmopolitan distributions (Corliss, 1979; Lynn, 2008). Hypotrichs are the most complex and highly differentiated group of ciliates, have a huge diversity with over 1,000 nominal species, and are abundant in waters (Berger, 1999, 2006, 2008, 2011; Foissner, 1999; Foissner, 2016; Song and Shao, 2017; Paiva, 2020; Shao et al., 2020; Wang et al., 2021). This group is playing an important role in the remineralization of organic material in aquatic ecosystems (Foissner, 1999). Besides, numerous recent studies have also focused on the classification and phylogeny of hypotrichs (Kaur et al., 2019; Park et al., 2020; Zhang et al., 2020; Chen et al., 2021; Jung et al., 2021; Li et al., 2021a,b; Luo et al., 2021; Omar et al., 2021a,b).

Stichotrichida is a group of hypotrichous ciliates that has always been difficult to classify. Its establishment is more due to the variable number and non-grouping of frontoventral-transverse cirral rows in its ciliary pattern (Berger, 2008; Lynn, 2008). Previous studies have shown that the dorsal infraciliature is at least as important as the ventral ciliary pattern for the estimation of the major phylogenetic relationships within the hypotrichs, and its application to the systematics of stichotrichids may prove to be particularly beneficial (Foissner et al., 2002; Berger, 2011).

The genus *Perisincirra* was erected by Jankowski (1978) with *Perisincirra kahli* (Grolière, 1975) Jankowski, 1978 as the type species. Unfortunately, some important morphological, morphogenetic, and molecular data are lacking for the type species *P. kahli*, so its diagnostic characters are insufficient for the systematics and classification of *Perisincirra* to be resolved. Thus, the inclusion of *Perisincirra longicirrata* Foissner et al., 2002 in this genus has been questioned. Here, the morphogenesis and phylogenetic position of *Perisincirra longicirrata* were investigated and discussed to determine whether it belongs to *Perisincirra* or if it represents a new genus.

## Materials and Methods

### Sample Site and Cultivation

On 12th September 2018, a mixture of water and silt was collected from a freshwater pond in Shenzhou Peninsula tourist resort (18°40′32.27′′N; 110°20′49.88′′E) in Wanning, China, when the water temperature was 25°C and the pH was about 7.0. The cells of *Pseudosincirra longicirrata* nov. comb. were isolated from the sample and were cultured at room temperature (25°C) in Petri dishes containing mineral water (Nongfu Spring) with rice grains added to promote the growth of bacteria as food for the ciliates. Although we failed to establish a clonal culture, there were no other hypotrichous morphospecies in the Petri dishes. Therefore, we are certain that the present morphological, morphogenetic, and molecular studies deal solely with *Pseudosincirra longicirrata* nov. comb.

### Morphology

Cells from the cultures were studied *in vivo* using bright-field and differential interference contrast microscopy at magnifications of 40–1,000 (Bai et al., 2020). The protargol (Sigma-Aldrich) silver staining method was used to reveal the infraciliature and nuclear apparatus (Wilbert, 1975). The drawings of stained specimens were made with a drawing device (Wu et al., 2020). To illustrate the changes occurring during morphogenetical processes, old (parental) ciliary structures are depicted by contour, whereas new ones are shaded black. Terminology follows Berger (2011).

### DNA Extraction, PCR Amplification, and Sequencing

Single cell of *Pseudosincirra longicirrata* nov. comb. was washed three times with sterilized water to remove contaminants and then transferred to a 1.5-ml microfuge tube with a minimum volume of water. Genomic DNA was extracted using a DNeasy Blood & Tissue Kit (Qiagen, Germany) following the manufacturer’s instructions. Small subunit ribosomal (SSU rDNA) amplification from the extracted DNA was carried out using primers 82S-F (5′-GAA ACT GCG AAT GGC TC-3′), 900F (5′-CGA TCA GAT ACC GTC CTA GT-3′), Pro B (5′-GGT TAA AAA GCT CGT AGT-3′), 900R (5′-ACT AGG ACG GTA TCT GAT CG-3′), and 18S-R (5′-GAT CCT TCT GCA GGT TCA CCT AC -3′). The conditions for PCR were as follows: denaturation at 98°C for 2 min; followed by 30 cycles of denaturation at 98°C for 10 s, annealing at 56°C for 15 s, extension at 72°C for 1 min 50 s, and a final extension step at 72°C for 7 min. Sequencing was performed bidirectionally by the Tsingke Biotechnology Co., Ltd. Xi’an Branch.

### Phylogenetic Analyses

The SSU rDNA sequence of *Pseudosincirra longicirrata* nov. comb. and those of 68 other hypotrichs downloaded from GenBank database were used for phylogenetic analyses. Four euplotid species were used as outgroup taxa (for accession numbers, see Figure 5). Sequences were aligned using the GUIDANCE web server^1^ (Penn et al., 2010). Maximum likelihood (ML) analyses were performed using RAxML-HPC2 on XSEDE v8.2.12 (Stamatakis et al., 2008) on the online server CIPRES Science Gateway^2^ (Miller et al., 2010). Bayesian inference (BI) analyses were carried out using MrBayes on XSEDE v3.2.7a (Ronquist et al., 2012) on CIPRES Science Gateway with the GTR + I + G model selected by Akaike information criterion (AIC) in MrModeltest v2 (Nylander, 2004). MEGA v5 was used to visualize the tree topologies (Tamura et al., 2011).

## Results

### ZooBank Registration

The ZooBank LSIDs are as follows:

Present work: urn:lsid:zoobank.org:pub:26F8BE7E-2AB4-40CB-AE5F-003D5019DBDB

*Pseudosincirra* nov. gen.: urn:lsid:zoobank.org:act: C46FEDCE-946F-4054-91CA-441CC0A42A68

*Pseudosincirra longicirrata* nov. comb.: urn:lsid:zoobank. org:act:C537D11B-3F9E-4366-B121-59FC1163FB28

### *Pseudosincirra* nov. gen.

#### Diagnosis

The new genus is diagnosed as follows: adoral zone of membranelles and undulating membranes is in *Gonostomum* pattern. There are three enlarged frontal cirri, one buccal cirrus, and one parabuccal cirrus. Postperistomial cirrus and transverse cirri are lacking. There are two long frontoventral rows and one right and two or more left marginal rows, and cirri within all rows are widely spaced. Dorsal kineties are in *Urosomoida* pattern, that is, three dorsal kineties and one dorsomarginal kinety. Caudal cirri are present.

#### Etymology

The name is a composite of *pseudo*- (false, i.e., resembling but not equaling) and suffix (-*sincirra*) of the genus name *Perisincirra* Jankowski, 1978. This indicates that *Pseudosincirra* has a cirral pattern similar to that of *Perisincirra* and has a feminine gender.

#### Type of Species

The type of species is *Perisincirra longicirrata* Foissner et al., 2002.

#### Remarks

*Pseudosincirra longicirrata* nov. comb. was previously assigned in *Perisincirra* as *Perisincirra longicirrata*. However, according to the origin of frontoventral-transverse cirral anlagen (FVT-anlagen) IV and V (for details, see *Divisional Morphogenesis*), it can be deduced that right marginal rows 1 and 2 of *Pseudosincirra longicirrata* nov. comb. described in Foissner et al. (2002) are actually frontoventral rows. It differs from the type species of *Perisincirra*, *P. kahli* (Grolière, 1975) Jankowski, 1978, in having two (vs. one) long ventral rows. Hence, a new genus, *Pseudosincirra* nov. gen., was erected.

### *Pseudosincirra longicirrata* (Foissner et al., 2002) nov. comb.

#### Improved Diagnosis

The size is 60–130 × 20–50 μm *in vivo*, with elongate ellipsoidal to bluntly fusiform. There are two macronuclear nodules and 18–26 adoral membranelles. There are two long frontoventral rows comprising 7–16 and 9–18 cirri, respectively. Three left marginal rows are composed of 5–14, 4–12, and 3–10 cirri, from inner to outer row, respectively. There is one right marginal row with 7–16 cirri. There are three frontal, one buccal, one parabuccal, and three caudal cirri. All the cirri are widely spaced and fine, up to 30-μm long.

#### Voucher Slides

Seven voucher slides (no. GQ2018091202A–G) with protargol-stained specimens were deposited in the Laboratory of Protozoological Biodiversity and Evolution in Wetland, Shaanxi Normal *University*, China.

#### Morphological Redescription

**FIGURE 1:**
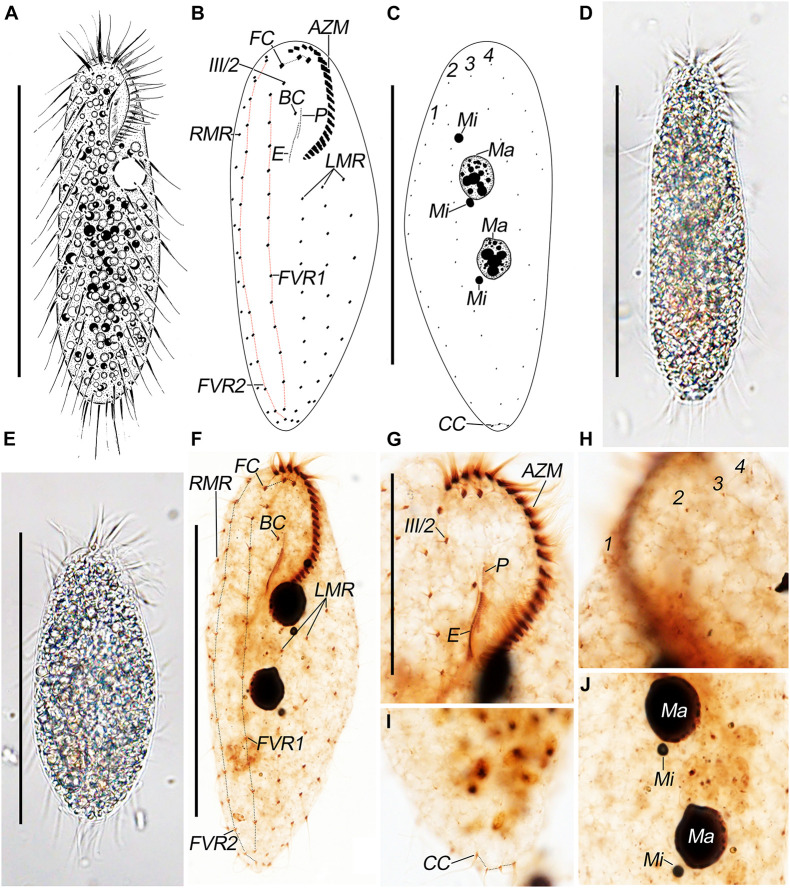
*Pseudosincirra longicirrata* nov. gen. and nov. comb. morphology. **(A)** Live and **(B,C)** after protargol staining. Photomicrographs of **(D,E)** live and **(F–J)** after protargol staining. **(A)** Ventral view of a representative individual. **(B,C)** Ventral **(B)** and dorsal **(C)** views to demonstrate the infraciliature. **(D,E)** Ventral views of representative individuals to show different body shapes. **(F)** Ventral view to demonstrate the infraciliature. **(G)** Ventral view of the anterior end of cell. **(H–J)** Dorsal views to demonstrate the dorsal kineties **(H)**, caudal cirri **(I)**, and nuclear apparatus **(J)**. AZM, adoral zone of membranelles; BC, buccal cirrus; CC, caudal cirri; E, endoral; FC, frontal cirri; FVR 1, 2, frontoventral rows 1, 2; III/2, cirrus III/2; LMR, left marginal row; Ma, macronuclear nodules; Mi, micronuclei; P, paroral; RMR, right marginal row; 1–3, dorsal kineties; 4, dorsomarginal kinety. Scale bars = 100 μm **(A–F)** and 40 μm **(G)**.

The body size is 90–130 × 30–50 μm *in vivo* (*n* = 16), usually about 120 × 40 μm; the length-to-width ratio is about 3:1 in live and 2–2.8:1, on average 2.5:1, in protargol preparations due to cell expansion caused by the fixative. The body is usually elongated ellipsoidal in shape, occasionally fusiform with the anterior region slightly more narrowed and the posterior portion indistinctly pointed (Figures 1A,D,E). The body is flexible but not contractile, only slightly flattened dorsoventrally. Invariably, two ellipsoidal macronuclear nodules, about 17 × 9 μm in size (after protargol staining), are located behind the buccal vertex near midline. Usually there are two, sometimes three, globular micronuclei about 2 μm in diameter, two of which are closely associated with macronuclear nodules (Figures 1C,J). A single contractile vacuole without distinct collecting canals, about 13 μm in diameter, is contracting at intervals of about 12 s, located about 33% down the length of the body near the left cell margin. Cortical granules are lacking. The cytoplasm is colorless, usually packed with highly refractive fat globules 1–6 μm across, making cells appear dark under low magnification. Crystals are sparse or lacking. Many individuals have food vacuole with orange contents on the right side of their bodies, about 20 μm across. Locomotion by slowly crawling on substrate was observed; when suspended, swimming while rotating about the longitudinal axis was observed.

The adoral zone of membranelles is about 27% of the body length *in vivo* and 30% on average in protargol preparations due to strong cell expansion caused by the fixative. Oral apparatus is in *Gonostomum* pattern. The adoral zone is composed of 22–26 membranelles (*n* = 16) with cilia up to 17-μm long. The endoral is located behind and parallel to the paroral and are almost equal in length (Figures 1B,F,G; Table 1).

**TABLE 1:** Morphometric characterization of the Chinese population of *Pseudosincirra longicirrata* nov. gen. and nov. comb.

**Character^a^**	**Min**	**Max**	**Med**	**Mean**	**SD**	**CV**	***n***
Body length	123	165	138	140.1	11.4	8.1	16
Body width	48	77	55	57.4	8.5	14.8	16
Body length-to-width ratio	2.0	2.8	2.5	2.47	0.21	8.65	16
Adoral zone of membranelle length	37	47	40	41.2	3.0	7.4	16
Adoral zone length-to-body length ratio	0.2	0.4	0.3	0.30	0.03	10.75	16
No. of adoral membranelles	22	26	24	23.8	0.9	3.9	16
No. of macronuclear nodules	2	2	2	2.0	0	0	16
Anterior macronuclear nodule length	13	21	17	17.3	2.4	13.6	16
Anterior macronuclear nodule width	7	11	8	8.8	1.5	17.2	16
No. of micronuclei	2	3	2	2.3	0.5	20.7	16
Micronuclei diameter	2	3	3	2.6	0.5	20.0	16
No. of frontal cirri	3	3	3	3.0	0	0	16
No. of buccal cirri	1	1	1	1.0	0	0	16
No. of parabuccal cirri	1	1	1	1.0	0	0	16
No. of frontoventral row	2	2	2	2.0	0	0	16
No. of cirri in frontoventral row 1	13	16	14	13.8	0.9	6.2	16
No. of cirri in frontoventral row 2	14	18	15	15.2	1.1	7.3	16
No. of left marginal rows	3	3	3	3.0	0	0	16
No. of cirri in inner left marginal row	10	13	12	11.3	1.1	9.5	16
No. of cirri in middle left marginal row	10	12	10	10.6	0.7	6.9	16
No. of cirri in outer left marginal row	7	9	8	8.1	0.6	7.1	16
No. of right marginal row	1	1	1	1.0	0	0	16
No. of cirri in right marginal row 1	12	16	14	13.6	1.2	8.5	16
No. of dorsal kineties	4	4	4	4.0	0	0	16
Bristle no. of dorsal kinety 1	12	14	13	13.2	0.8	5.7	16
Bristle no. of dorsal kinety 2	12	16	15	14.7	1.2	8.1	16
Bristle no. of dorsal kinety 3	11	13	13	12.3	0.8	6.4	16
Bristle no. of dorsal kinety 4	5	8	6	6.1	1.0	16.7	16
No. of caudal cirri	3	4	3	3.2	0.4	12.6	16

*CV, coefficient of variation in percent; Max, maximum; Mean, arithmetic mean; Med, median; Min, minimum; *n*, sample size; no., number; SD, standard deviation. ^*a*^All data are based on protargol-stained specimens with measurements in micrometers.*

The ciliary pattern on the ventral side is rather constant although the number of cirri within rows is variable. All cirri are rather long and conspicuous, with cilia of the frontal cirri and the marginal cirri about 20-μm long. Constantly, there are three frontal cirri with the rightmost one in front of cirrus III/2. Buccal cirrus is ahead of the endoral and slightly behind the anterior end of the paroral. There are two long frontoventral rows; the inner row (FVR1) is composed of 13–16 cirri and commences slightly behind the level of cirrus III/2, and the outer row (FVR2) consists of 14–18 cirri and commences at the approximately same level as the rightmost frontal cirrus; both rows terminate at the posterior end of the cell. There are three widely spaced left marginal rows, commencing near the proximal end of the adoral zone, composed of 10–13, 10–12, and 7–9 cirri, from inner to outer row, respectively. Invariably, there is one right marginal row, with 12–16 cirri, which is almost bipolar (Figures 1B,F,G). All cirri are fine, and most frontoventral and marginal cirri are composed of four basal bodies and widely spaced.

Dorsal bristles are about 5-μm long; constantly arranged in four kineties; and composed of 12–14, 12–16, 11–13, and 5–8 dikinetids, respectively. Dorsal kineties 2 and 3 are almost bipolar; kinety 1 commences about 20% down the length of the body; kinety 4 (dorsomarginal kinety) terminates about 35% down the length of body. Usually, there are three caudal cirri, one at the posterior end of each of dorsal kineties 1–3; sometimes, there are four caudal cirri (in about 25% of the individuals), two at the posterior end of dorsal kinety 1 and one at the posterior end of each of dorsal kineties 2 and 3 (Figures 1C,H,I).

### Divisional Morphogenesis

#### Development of the Ventral Ciliature

**FIGURE 2:**
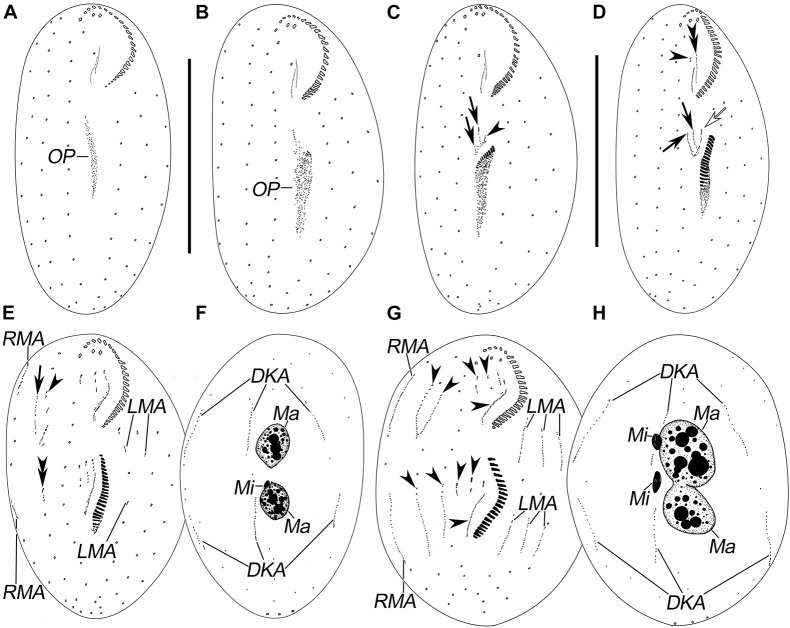
Morphogenesis of *Pseudosincirra longicirrata* nov. gen., nov. comb. after protargol staining. **(A,B)** Ventral views of very early dividers showing the newly formed oral primordium. **(C)** Ventral view of an early divider; arrows mark the FVT-anlagen and arrowhead points to the undulating membranes anlage. **(D)** Ventral view of a slightly later phase; arrows display the FVT-anlagen, hollow arrow denotes the undulating membrane anlage, and arrowhead and double arrowhead mark the dedifferentiation of buccal cirrus and the parental paroral and endoral, respectively. **(E,F)** Ventral and dorsal views of a later divider; the arrow shows anlage V in the proter, arrowhead denotes anlage IV in the proter, and double arrowhead marks anlage V in the opisthe. **(G,H)** Ventral and dorsal views of a cell in prometaphase; arrowheads demonstrate the anlagen I–V in the proter and opisthe, respectively. DKA, dorsal kinety anlagen; FVT-anlagen, frontoventral-transverse cirral anlagen; LMA, left marginal anlagen; Ma, macronuclear nodules; Mi, micronuclei; OP, oral primordium; RMA, right marginal anlagen. Scale bars = 100 μm.

The oral primordium for the opisthe appears in the postoral area between the inner left marginal row and frontoventral row 1. With the proliferation of basal bodies, the oral primordium lengthens posteriorly and differentiates posteriad (Figures 2A–D, 4A,B). Three streaks are formed anteriorly to the right of the oral primordium (Figures 2C,D,  4C,E).

In the proter, the parental adoral zone remains intact. Anlage I forms from the dedifferentiated parental paroral and endoral, anlage II develops from the dedifferentiated parental buccal cirrus, and anlage III is generated from the dedifferentiated parental cirrus III/2 (as shown in Figures 2D,E, 4D). Up to this point, three FVT-anlagen are formed in each filial product.

**FIGURE 3:**
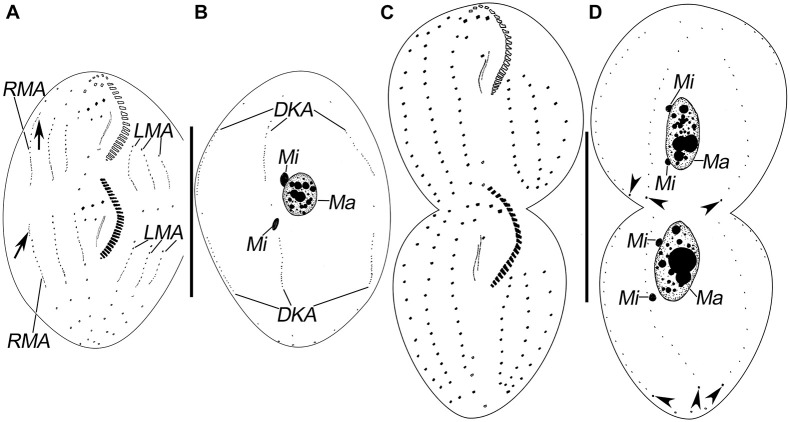
Morphogenesis of *Pseudosincirra longicirrata* nov. gen. and nov. comb. after protargol staining. **(A,B)** Ventral and dorsal views of a middle divider; arrows show the dorsomarginal kinety anlagen. **(C,D)** Ventral and dorsal views of a late divider to show the infraciliature; arrowheads point to the caudal cirri. DKA, dorsal kinety anlagen; LMA, left marginal anlagen; Ma, macronuclear nodules; Mi, micronuclei; RMA, right marginal anlagen. Scale bars = 100 μm.

**FIGURE 4:**
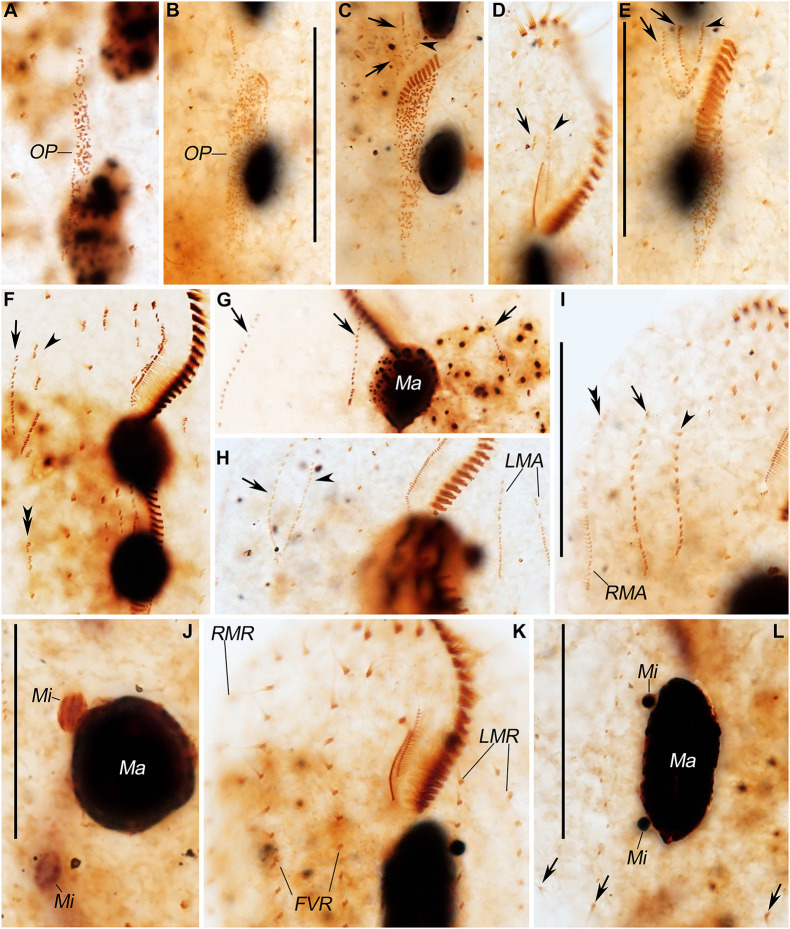
Photomicrographs of *Pseudosincirra longicirrata* nov. gen. and nov. comb. during morphogenesis after protargol staining. **(A,B)** Ventral views of very early phases showing the newly formed oral primordium. **(C)** Ventral view of an early divider; arrows display the FVT-anlagen, while arrowhead marks the undulating membrane anlage. **(D,E)** Ventral views of a slightly later divider. In panel **(D)**, the arrow and arrowhead mark the dedifferentiation of buccal cirrus and the parental paroral and endoral, respectively. Arrows and arrowhead in panel **(E)** show the FVT-anlagen and undulating membrane anlage, respectively. **(F,H,I)** Ventral views; arrows mark anlage V in the proter, arrowheads denote anlage IV in the proter, double arrowhead in panel **(F)** reveals anlage V in the opisthe, and double-arrowhead in panel **(I)** points to the dorsomarginal kinety anlage. **(G)** Dorsal view; arrows demonstrate the dorsal kinety anlagen. **(J)** Dorsal view of the same cell as shown in panel **(I)**, showing the fusion of the macronuclear nodules. **(K,L)** Ventral and dorsal views of a late divider, arrows in panel **(L)** indicate the newly formed caudal cirri. FVR, frontoventral rows; FVT-anlagen, frontoventral-transverse cirral anlagen; LMA, left marginal anlagen; LMR, left marginal row; Ma, macronuclear nodules; Mi, micronuclei; OP, oral primordium; RMA, right marginal anlagen; RMR, right marginal row. Scale bars = 40 μm.

Soon after, in the proter, anlage IV is generated within the frontoventral row 2, and anlage V develops *de novo* to the right of frontoventral row 2. In the opisthe, anlagen IV and V are generated within frontoventral rows 1 and 2, respectively (Figures 2E,G, 4F). Then, in the proter, anlagen IV and V migrate to the mid-region of the body (Figures 2G, 3A,  4H,I).

Finally, anlage I produces the left frontal cirrus and undulating membranes, anlage II generates the middle frontal cirrus and buccal cirrus, anlage III develops into the right frontal cirrus and cirrus III/2, while anlagen IV and V generate frontoventral rows 1 and 2, respectively (Figures 3A,C, 4K).

#### Development of Marginal Rows and Dorsal Kineties

The marginal row anlagen and dorsal kinety anlagen develop intrakinetally within the parental marginal rows and dorsal kineties 1–3 in each daughter cell, respectively. These anlagen enlarge by proliferation of basal bodies and stretch in both directions to eventually replace the parental rows (Figures 2E–H, 3A,B, 4G–I). In addition, a short streak of basal bodies, i.e., the dorsal kinety 4 anlage, develops ahead of the anteriormost portion of the right marginal anlage in both the proter and the opisthe (Figures 3A, 4I). One or two caudal cirri are formed at the posterior end of dorsal kinety anlage 1, and constantly, one caudal cirrus is formed at the posterior end of each dorsal kineties anlagen 2 and 3 (Figures 3D, 4L).

#### Division of Nuclear Apparatus

In this process, the two macronuclear nodules fuse into a single mass (Figures 3B, 4J). At later stages of morphogenesis, the mass splits and is distributed between the proter and the opisthe (Figures 3D, 4L). The micronuclei divide mitotically (Figures 2H, 3B,D, 4J,L).

#### Phylogenetic Analyses Based on SSU rDNA Gene Sequences

**FIGURE 5:**
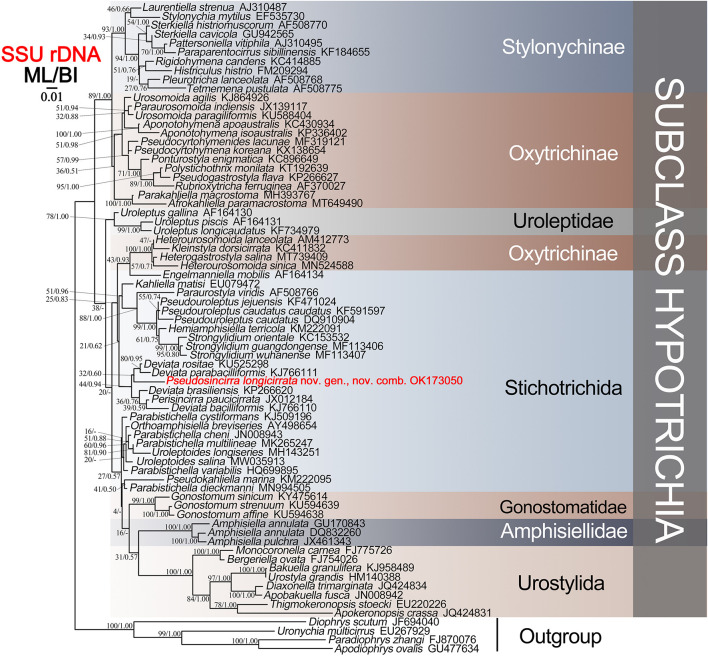
Maximum likelihood (ML) tree based on the SSU rDNA sequence data. The newly sequenced *Pseudosincirra longicirrata* nov. gen. and nov. comb. are indicated in red. Numbers at the nodes represent the bootstrap values of ML and the posterior probabilities of Bayesian analysis (BI), respectively. A hyphen “-” indicates the disagreement between the BI tree and the reference ML tree. All branches are drawn to scale. The scale bar corresponds to 0.01 expected substitutions per site.

The SSU rDNA sequence of *Pseudosincirra longicirrata* nov. comb. was deposited in GenBank with the accession number OK173050. The length and GC content of the new sequence are 1,613 bp and 45.38%, respectively. Since the topologies of ML tree and BI tree are basically the same, only the ML tree is shown with nodal support for both algorithms (Figure 5).

The phylogenetic analyses showed that *Pseudosincirra longicirrata* nov. comb. groups with *Deviata baciliformis* (Gelei, 1954) Eigner, 1995; *D. brasiliensis* Siqueira-Castro et al., 2009; *D. parabaciliformis* Li et al., 2014; *D. rositae* Küppers et al., 2007; and *Perisincirra paucicirrata* Foissner et al., 2002. The SSU rDNA sequence similarities of *Pseudosincirra longicirrata* nov. comb. to *D. baciliformis*, *D. brasiliensis*, *D. parabaciliformis*, *D. rositae*, and *P. paucicirrata* are 94.2, 93.2, 94.3, 91.5, and 91.2%, respectively.

## Discussion

### Establishment of the New Genus

In terms of having three more or less long cirral rows right in the cell midline, two or more cirral rows left of the cell midline, and a dorsomarginal kinety, *Pseudosincirra* nov. gen. should be compared with six genera, namely, *Afrokahliella* Berger, 2011; *Fragmocirrus* Foissner, 2000; *Idiodeviata* Foissner, 2016; *Kahliella* Corliss, 1960; *Neogeneia* Eigner, 1995; and *Parakahliella* Berger et al., 1985.

The new genus differs from *Afrokahliella* in having a *Gonostomum*-patterned (vs. *Oxytricha*-patterned) adoral zone of membranelles, fine (vs. moderately thick) cirri, very widely (vs. narrowly) spaced cirri in all rows, and caudal cirri at the end of dorsal kineties 1–3 (vs. at the end of dorsal kineties 1 and 2 only) (Berger, 2011).

Compared with *Fragmocirrus*, the new genus has a *Gonostomum*-patterned (vs. *Oxytricha*-patterned) adoral zone of membranelles and undulating membranes, a single buccal cirrus and a single parabuccal cirrus (vs. a buccal row and a parabuccal row), transverse cirri absent (vs. present), fine cirri (vs. moderately thick), cirri within all rows very widely (vs. narrowly) spaced, and caudal cirri at the end of dorsal kineties 1–3 (vs. at the end of dorsal kineties 1 and 2 only) (Berger, 2011).

*Pseudosincirra* nov. gen. differs from *Idiodeviata* in the presence (vs. absence) of caudal cirri and in having three (vs. one) dorsal kineties (Foissner, 2016).

*Kahliella* resembles *Pseudosincirra* nov. gen. in terms of the structure of its adoral zone of membranelles and undulating membranes, but can be separated from the latter by having three (vs. two) frontoventral rows, moderately thick (vs. fine) cirri and cirri within all rows narrowly (vs. widely) spaced, parental left marginal rows retained (vs. resorbed) in postdividers, and caudal cirri absent (vs. present) (Berger, 2011).

*Pseudosincirra* nov. gen. can be separated from *Neogeneia* by having a *Gonostomum*-patterned (vs. *Oxytricha*-patterned) adoral zone of membranelles, fine (vs. moderately thick) cirri, and parental right and left marginal cirri not retained (vs. retained) in postdividers (Berger, 2011).

*Pseudosincirra* nov. gen. differs from *Parakahliella* in having a *Gonostomum*-patterned (vs. *Oxytricha*-patterned) adoral zone of membranelles and undulating membranes, fine (vs. moderately thick) cirri, all rows with very widely (vs. narrowly) spaced cirri, a single buccal and a single parabuccal cirrus (vs. a buccal row and a parabuccal row), parental dorsal kineties absorbed (vs. retained) in postdividers, and caudal cirri at the end of dorsal kineties 1–3 (vs. at the end of dorsal kineties 1 and 2 only) (Berger, 2011).

*Pseudosincirra* nov. gen. resembles *Perisincirra* in having widely spaced cirri in all rows, but can be separated from the latter by having two (vs. one) long ventral rows (Berger, 2011).

### Identification of the Chinese Population of *Pseudosincirra longicirrata* nov. comb. and Comparison With Two African Populations

*Pseudosincirra longicirrata* nov. comb. was first reported by Foissner et al. (2002) as *Perisincirra longicirrata* based on populations discovered in Benin and Namibia. The present population differs from the Benin and Namibian populations in having the following: more adoral membranelles (22–26 vs. 18–22); a smaller ratio of adoral zone length to body length (24–37% and average 30 vs. 29–44% and average 37%); more cirri in the frontoventral rows 1 and 2 (13–16 and 14–18 vs. 7–12 and 9–14) and in the middle left marginal row (10–12 vs. 4–10); usually three, sometimes four, caudal cirri (vs. invariably three caudal cirri); two or three micronuclei (vs. one or two micronuclei); and shorter cilia on the ventral side (20 vs. 30-μm long). However, we consider these differences to be population-dependent and therefore not significant for species-level separation. The identity of the present population is therefore not in doubt.

### Morphogenesis

Previous studies on *Pseudosincirra longicirrata* nov. comb. were limited to morphology, so this is the first report of its morphogenesis (Foissner et al., 2002). According to the origin of FVT-anlagen IV and V in the proter, it can be deduced that right marginal rows 1 and 2 described in Foissner et al. (2002) are actually frontoventral rows. Hence, only one right marginal row is present. Considering the formative mode of FVT-anlagen IV and V, we compare *P. longicirrata* nov. comb. with some similar species that possess three clearly differentiated frontal cirri and at least two more or less long frontoventral rows and whose FVT-anlagen development is well known, i.e., *Afrokahliella paramacrostoma* Li et al., 2021a; *Deviata abbrevescens* Eigner, 1995; *D. baciliformis*; *D. brasiliensis*; *D. parabaciliformis*; *Fragmocirrus espeletiae* Foissner, 2000; *Khaliella simplex* (Horváth, 1934) Berger, 2011; *Neogeneia hortualis* Eigner, 1995; and *Parakahliella macrostoma* (Foissner, 1982) Berger et al., 1985 (for details, see Table 2). In all these genera, not all the FVT-anlagen IV–VI in both daughter cells develop within the corresponding parental frontoventral rows IV–VI, as in *Parastrongylidium oswaldi* (Aescht and Foissner, 1992). We speculated that this point may be phylogenetically informative pending greater taxon sampling and the availability of further information on molecular phylogeny of these species.

**TABLE 2:** Morphogenetic comparison of the Chinese population of *Pseudosincirra longicirrata* nov. gen. and nov. comb. with similar species showing the origin of frontoventral-transverse cirral anlagen.

	**Proter**						**Opisthe**						
	**I**	**II**	**III**	**IV**	**V**	**VI**	**I**	**II**	**III**	**IV**	**V**	**VI**	
*Pseudosincirra longicirrata*	PUM	BC	PBC	FVR V	*De novo*		OP	OP	OP	FVR IV	FVR V		
*Afrokahliella paramacrostoma*	PUM	BC	PBC	*De novo*	FVR V		–	–	–	–	FVR V		Li et al., 2021a
*Deviata parabaciliformis*	PUM	BC	PBC	FVR IV	FVR VI	*De novo* or anlage VI for opisthe	OP	OP	OP	FVR V	*De novo*	FVR VI	Li et al., 2014
*Deviata baciliformis*	PUM	BC	PBC	FVR IV	FVR VI?		OP	OP	OP	FVR V	*De novo*		Berger, 2011; Li et al., 2015;
*Deviata abbrevescens*	PUM	BC	PBC	FVR IV and V	FVR V and VI	FVR VI	OP	OP	OP (and FVR IV?)	FVR V	FVR V and VI	FVR VI	Eigner, 1995
*Deviata brasiliensis*	PUM	BC	PBC	FVR IV	FVR V	FVR VI	OP	OP	FVR IV	FVR V	FVR V or VI	FVR VI	Siqueira-Castro et al., 2009: Luo et al., 2016
*Fragmocirrus espeletiae*	PUM	BC	PBC	FVR V	FVR V		OP	OP	OP	FVR IV	FVR IV		Foissner, 2000
*Khaliella simplex*	PUM?	BC	PBC	FVR IV	FVR V	FVR V	OP	OP	OP or FVR IV and V	FVR IV?	FVR V	FVR V	Fleury and Fryd-Versavel, 1982; Eigner, 1995
*Neogeneia hortualis*	PUM	BC	PBC	FVR IV	FVR V		OP	OP	OP	FVR IV	*De novo*		Eigner, 1995
*Parakahliella macrostoma*	PUM	BC	PBC	FVR V	FVR V		OP and FVR IV	OP	OP	FVR IV	FVR V		Berger et al., 1985

*BC, buccal cirrus (i); FVR IV–VI, frontoventral rows IV–VI; I–VI, frontoventral-transverse cirral anlagen I–VI; OP, oral primordium; PBC, parabuccal cirri/cirrus III/2; PUM, parental undulating membranes; ?, uncertain.*

### Molecular Phylogeny

*Pseudosincirra longicirrata* nov. comb. falls in the *Deviata baciliformis + D. brasiliensis + D. parabaciliformis + D. rositae + Perisincirra paucicirrata* clade, the close relationship between these six species being supported by having fine cirri, i.e., cirri in the ventral and marginal rows are mostly composed of two or four cilia and cirri within all rows are relatively widely spaced. The presence or absence of dorsomarginal kineties and the number of dorsal kineties vary in these species and other species in the family Deviatidae Foissner, 2016. We agree with Foissner (2016) that “the feature of presence/absence of dorsomarginal kineties evolved several times independently” and posit that *Pseudosincirra* and *Perisincirra paucicirrata* should be assigned to Deviatidae. Furthermore, we suggest that the possession of fine cirri and the relatively widely spaced cirri within all rows should be considered as plesiomorphies of this family/group and added in the diagnosis of the family.

Some species of the family Deviatidae cluster with *Strongylidium*–*Hemiamphisiella*–*Pseudouroleptus*, which is close to Dorsomarginalia and Dorsomarginalian species in present and previous studies (Lu et al., 2020; Wang et al., 2020; Ma et al., 2021; Vd’ačný and Foissner, 2021). Hence, we disagree with Foissner (2016) that Deviatidae is possibly sister to the non-dorsomarginalian Kahliellidae Tuffrau, 1979, but closely related to Dorsomarginalia or *Strongylidium*–*Hemiamphisiella*–*Pseudouroleptus.*

## Data Availability Statement

The data presented in the study are deposited in the GenBank database, accession number OK173050.

## Author Contributions

QG and QT collected the samples and carried out almost all of the experiments (preparations, illustrations, micrographs, etc.). All authors did the identification of the species and wrote the manuscript. All authors contributed to the article and approved the submitted version.

## Conflict of Interest

The authors declare that the research was conducted in the absence of any commercial or financial relationships that could be construed as a potential conflict of interest.

## Publisher’s Note

All claims expressed in this article are solely those of the authors and do not necessarily represent those of their affiliated organizations, or those of the publisher, the editors and the reviewers. Any product that may be evaluated in this article, or claim that may be made by its manufacturer, is not guaranteed or endorsed by the publisher.

## Footnotes

1
http://guidance.tau.ac.il/

2
http://www.phylo.org/portal2/login!input.action
